# Laboratory and Neuroimaging Biomarkers in Neuropsychiatric Systemic Lupus Erythematosus: Where Do We Stand, Where To Go?

**DOI:** 10.3389/fmed.2018.00340

**Published:** 2018-12-04

**Authors:** César Magro-Checa, Gerda M. Steup-Beekman, Tom W. Huizinga, Mark A. van Buchem, Itamar Ronen

**Affiliations:** ^1^Department of Rheumatology, Leiden University Medical Center, Leiden, Netherlands; ^2^Department of Rheumatology, Zuyderland Medical Center, Heerlen, Netherlands; ^3^Department of Radiology, Leiden University Medical Center, Leiden, Netherlands; ^4^Department of Radiology, C.J. Gorter Center for High Field MRI, Leiden University Medical Center, Leiden, Netherlands

**Keywords:** systemic lupus erythematosus, neuropsychiatric systemic lupus erythematosus, NP-SLE, neuroimaging, magnetic resonance imaging, biomarkers

## Abstract

Systemic lupus erythematosus (SLE) is a chronic autoimmune disease characterized by multi-systemic involvement. Nervous system involvement in SLE leads to a series of uncommon and heterogeneous neuropsychiatric (NP) manifestations. Current knowledge on the underlying pathogenic processes and their subsequent pathophysiological changes leading to NP-SLE manifestations is incomplete. Several putative laboratory biomarkers have been proposed as contributors to the genesis of SLE-related nervous system damage. Alongside the laboratory biomarkers, several neuroimaging tools have shown to reflect the nature of tissue microstructural damage associated with SLE, and thus were suggested to contribute to the understanding of the pathophysiological changes and subsequently help in clinical decision making. However, the number of useful biomarkers in NP-SLE in clinical practice is disconcertingly modest. In some cases it is not clear whether the biomarker is truly involved in pathogenesis, or the result of non-specific pathophysiological changes in the nervous system (e.g., neuroinflammation) or whether it is the consequence of a concomitant underlying abnormality related to SLE activity. In order to improve the diagnosis of NP-SLE and provide a better targeted care to these patients, there is still a need to develop and validate a range of biomarkers that reliably capture the different aspects of disease heterogeneity. This article critically reviews the current state of knowledge on laboratory and neuroimaging biomarkers in NP-SLE, discusses the factors that need to be addressed to make these biomarkers suitable for clinical application, and suggests potential future research paths to address important unmet needs in the NP-SLE field.

## Introduction

**Figure 1:**
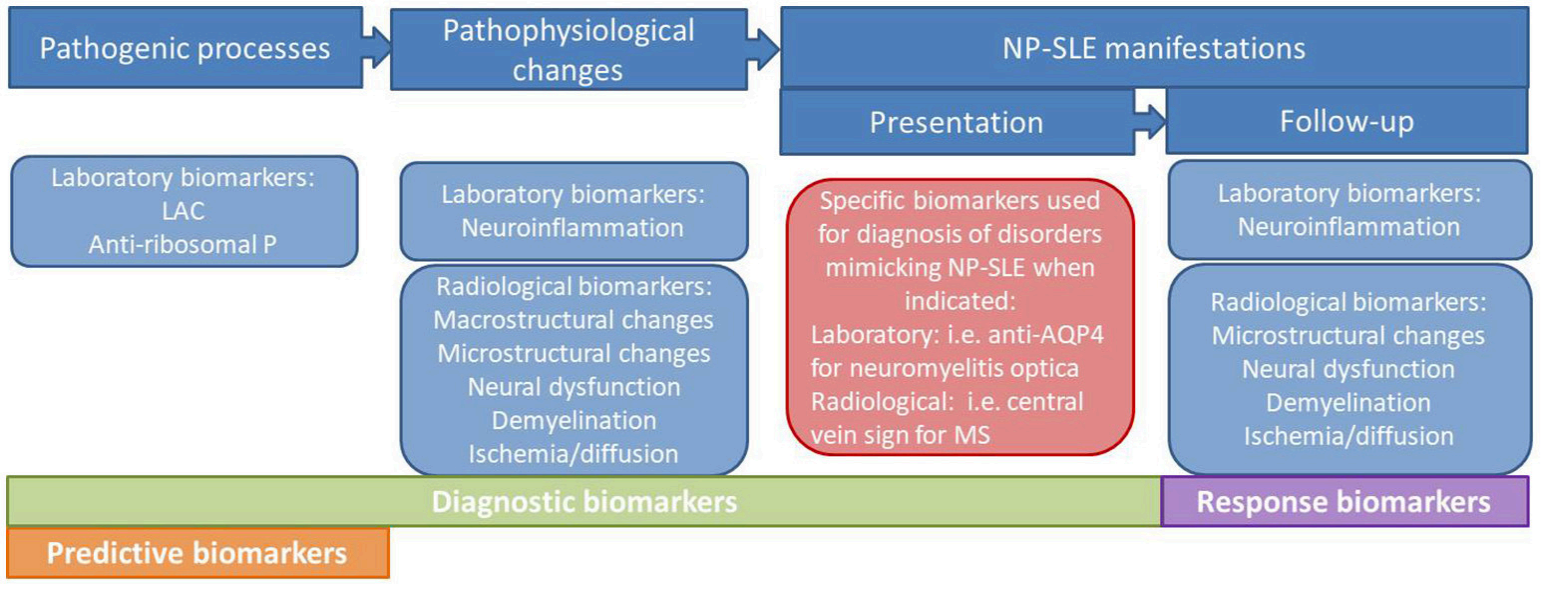
Types of biomarkers in NP-SLE.

For the last several years, clinicians and researchers in the field of neuropsychiatric systemic lupus erythematosus (NP-SLE) have been emphasizing the need for standardized and validated biomarkers to be used in clinical practice. Dozens of putative molecular mechanisms, such as serum and cerebrospinal fluid (CSF) antibodies to neuronal cell and cellular components, cytokines, complement and other immunochemical phenomena have been proposed to play a role in the genesis of nervous system involvement in SLE (1). Moreover, several neuroimaging techniques such as magnetic resonance imaging (MRI) and nuclear medicine techniques have allowed the characterization of structural and functional abnormalities in SLE patients therefore helping to better understand the underlying pathogenesis and subsequent pathophysiological changes (2, 3). Ideally, these laboratory and neuroimaging biomarkers, or a combination of them, would be used in clinical practice for the attribution of NP symptoms to SLE, as well as prognostic factors, predictors of response to therapies or even to develop new targeted therapies (Figure 1) (2). Despite these efforts, so far none of the postulated biomarkers has been demonstrated reproducible or ubiquitous enough to become a specific biomarker for NP-SLE. In clinical practice, the presentation of NP symptoms in SLE patients still poses an important diagnostic and therapeutic challenge to the physician. So far, the best strategy for diagnosing NP-SLE remains multidisciplinary expert consensus after standardized assessment (4, 5). An important goal of ongoing research is the development and validation of a range of laboratory and imaging biomarkers that reliably capture the different aspects of nervous system involvement in SLE and enable clinicians to attribute these symptoms either to SLE or to other etiologies.

This review compiles current knowledge on candidate biomarkers on NP-SLE. We discuss the strength of evidence for proposed laboratory and neuroimaging biomarkers; hypothesize about the desired properties of a biomarker in NP-SLE; and comment about the obstacles for biomarker development and its application in clinical practice. We conclude by highlighting some promising avenues for such biomarkers and suggest some strategies to increase their specificity and therefore increase their diagnostic role in clinical practice.

## What We Have Learned From Animal Studies

Most if not all current knowledge about NP-SLE pathogenesis comes from mice models. Passive-transfer experiments in mice, consisting of injection of a certain substance or autoantibody, are the most common preclinical disease model in NP-SLE. Three different families of SLE mice models are currently used: induced models, spontaneous models and genetically engineered knockout and transgenic models. In the case of spontaneous models, these mice spontaneously manifest over varying periods of time with a series of clinical and serological features comparable to human SLE. NP manifestations are only described to appear in these models. Several lupus-prone strains have been so far the most commonly used for modeling NP-SLE manifestations: MRL/lpr, NZB/NZW F1 and 564Igi mice models (Panel 1 in Supplementary Material) (6, 7).

Studies using these mice models have provided some insight into the underlying pathogenic mechanisms contributing to NP-SLE in humans. Autoantibodies against neuronal auto-antigens, as well as other molecules are among the proposed mechanisms:

- *Anti-ribosomal P (RP) antibody*: Several studies have tried to explain the neuro-pathogenic effect of this antibody. After injection into the ventricles or hippocampus in mice, anti-RP antibodies induced depression-like behavior and memory impairment in mice, respectively, and were found to target the limbic system, especially the neurons in the hippocampus, cingulate cortex, and the primary olfactory piriform cortex (7, 8). Segovia-Miranda et al. have proposed that anti-RP targets the neuronal cell surface P antigen, or NSPA, in specific brain regions. This interaction may alter glutamatergic synaptic transmission in the hippocampus by involving both α-amino-3-hydroxy-5-methyl-4-isoxazolepropionic acid receptor (AMPAR) and N-methyl-D-aspartate receptor (NMDAR) activation and compromise synaptic plasticity involved in memory, hypothetically leading to cognitive dysfunction and other diffuse NP manifestations (9).
- Antibodies against the NMDAR subtypes 2a and 2b (*anti-NR2 antibodies*): Previous studies by Diamond *et al* have demonstrated how a murine monoclonal anti-dsDNA antibody cross-reacts with an amino-acid present in the subunits NR2a and NR2b of NMDAR, and how injecting these antibodies into mice leads to hippocampal neuronal death by apoptosis and cognitive impairment (10). Anti-NR2 antibodies have been also related to neuronal dysfunction and death in the hippocampus and amygdala in MRL/lpr mice (11). However, the mere presence of anti-NR2 antibodies in the blood of mice does not lead to neural death or subsequent NP symptoms; it is proposed in this work that to exert an effect upon neurons, anti-NR2 antibodies need to gain access to the brain through a disrupted blood brain barrier (BBB) (12, 13). Once these antibodies reach the brain they may produce several interactions. The acute exposure to the NMDAR may depend on the dose: at low concentrations they alter synaptic function; higher concentrations lead to excessive NMDAR activation causing neuronal cell death by apoptosis. Furthermore, chronic irreversible functional and structural damage of surviving neurons has been described to persist even when antibodies are no longer present. Impaired memory and hippocampal atrophy, as well as emotional disturbances and atrophy of the amygdala have been described to follow in mice (14, 15).
- An anti-dsDNA idiotype (Id) antibody in SLE, *16/6-Id antibody*, hampers visual-recognition and results in cognitive impairments via cross-reaction with cytoskeletal proteins, glycoproteins and brain glycolipids. Kivity et al. showed an increased number of activated astrocytes and microglial cells as markers of brain inflammation after ventricular injection of this antibody in mice (16).
- *Neurofilament alpha-internexin (INA)*: A murine model developed by INA immunization demonstrated cortical and hippocampal neuron apoptosis that resulted in pronounced cognitive dysfunction and memory loss (17).

Other molecules have been more recently also involved in NP-SLE pathogenesis in mice:

- *Interferon (IFN) alpha*: This inflammatory mediator has been postulated as one of the most promising targets in NP-SLE. Recently, Bialas *et al* have demonstrated the way in which peripheral type I INF enters the brain of 5641gi and NZB/NZW F1 strains and stimulates microglial engulfment of synaptic material. It was shown how reactive microglia lead to synapse loss in the frontal cortex of these mice, and these findings correlated with the appearance of behavioral deficits. Furthermore, it was shown how targeting the INF with anti-IFNAR antibodies prevented these symptoms and also mitigated synapse loss and microglial dysfunction (18).
- *Complement cascade*: Complement has been proposed as a candidate mechanism participating in microglial engulfing of synapses in the lupus brain. Recent discoveries on mouse models of Alzheimer's disease confirmed a critical role of the classical complement cascade in early synapse loss (19, 20). Other alternative role of complement is the regulation of brain inflammation. Alexander et al. showed that the deletion of a key alternative pathway protein known as factor B, and the inhibition of the classical and alternative complement cascades with the soluble complement inhibitor Crry-Ig alleviated NP symptoms of MRL-lpr mice (21, 22). Ulterior studies of the same group using the same mice model showed that selective inhibition of complement receptors C3aR and C5aR resulted, respectively in a reduction in neuronal degeneration and a drop in NP symptoms (23, 24). The same group also showed that C5 plays an important role in the maintenance of BBB in MRL-lpr mice (25).
- *TWEAK*: Apart from the alternative complement cascade, other molecules have shown to be important modulators of the integrity of the BBB and subsequently the transport into and out of the brain parenchyma (26). A pro-inflammatory cytokine member of the TNF superfamily called TWEAK (TNF-like weak inducer of apoptosis) induced cellular proliferation, angiogenesis, apoptosis, and production of inflammatory cytokines and chemokines through its receptor Fn14 (27). MRL/lpr mice lacking Fn14 improved cognitive function and exhibited less depression symptoms such as anhedonia (28).

## Laboratory Biomarkers in Human NP-SLE: Looking for a Crystal Ball?

In 1998, The National Institutes of Health Biomarkers Definitions Working Group established by consensus the definition of a biomarker as “a characteristic that is objectively measured and evaluated as an indicator of normal biological processes, pathogenic processes or pharmacological responses to a therapeutic intervention”(29). This definition was later expanded by the World Health Organization (WHO) through the International Program of Chemical Safety to include “any substance, structure, or process that can be measured in the body or its products and influence or predict the incidence of outcome or disease” (30). This definitions are mainly applicable for laboratory-measured biomarkers. The first step in the path of biomarker development is invariably the identification and proposition as candidate of a marker of disease or any of its manifestations (*exploratory biomarker*). Subsequently, the reproducibility of findings in other studies across different populations will test the actual effectiveness of this biomarker (*validated biomarker*). Ultimately, these biomarkers may become clinically relevant (*clinically useful biomarker*) (31). In multiple sclerosis (MS), a neurological disease sharing some similarities with NP-SLE, biomarker research has significantly progressed in the last years; biomarkers for MS have been categorized into predictive, diagnostic, disease activity and treatment-response biomarkers (32). In current medical literature we find many studies directly identifying new potential biomarkers associated with NP-SLE or a specific neuropsychiatric symptom (e.g., cognitive dysfunction) due to SLE. Production of autoantibodies is a hallmark of SLE, with around 200 different antibodies more often present in SLE than in controls described so far (33). Based on data from the mouse models mentioned above, it is reasonable to assume that the target of certain antigens in the central nervous system by one or more antibodies may play a causal role in the genesis of NP-SLE. In humans, the concept that serum antibodies can lead to neurological symptoms is not new and auto-immune limbic encephalitis (LE) is a good example for that. LEs are associated with three neuronal cell-surface antibodies targeting leucine-rich glioma-inactivated 1 (anti-LGI1), γ-amino-butyric acid B-receptor (anti-GABAbR), and anti-AMPAR, usually leading to irreversible neurological deficits by altering the structure or function of the target antigen (34). In NP-SLE, several auto-antibodies targeting antigens located in brain tissue, as well as auto-antibodies directed against ubiquitous cellular components have been proposed as exploratory biomarkers (Table 1). Most studies report a higher proportion of positivity for a certain antibody in the serum or CSF in NP-SLE patients when compared with SLE and suggest a role for that antibody as diagnostic biomarker. An important number of these antibodies have only been found in discovery studies while reproducibility of findings across different patient populations is lacking. Furthermore, contradictory results among studies are common. In other cases the association has not been found in reproducibility studies (Supplementary Table 1); therefore so far only few antibodies have been validated as biomarkers. In a prospective study including 1,047 SLE patients, Hanly et al. found a role for lupus anticoagulant (LAC) and for anti-ribosomal P as predictive biomarkers for intracranial thrombosis and lupus psychosis, respectively (35). Three meta-analyses evaluating the role of antibodies as diagnostic biomarkers in NP-SLE have been published so far (36–38). Compared with SLE, NP-SLE patients had higher proportion of elevated serum levels of anticardiolipin, LAC, anti-ribosomal P and anti-neuronal antibodies, and an increased prevalence of positive titers for CSF anti-neuronal antibodies (37). These results are conflicting with a previous meta-analysis that combined data from 1,537 SLE patients from 14 centers and suggested a limited value for anti-P ribosomal antibodies as diagnostic biomarker in NP-SLE or any of its manifestations (38). Also the role of serum anti-NMDAR antibodies has been evaluated in a meta-analysis that included data from a total of 2,212 SLE patients. A higher positivity for serum anti-NMDAR was found in NP-SLE compared with SLE; this study concluded that serum anti-NMDAR antibodies may have a diagnostic value collectively but cannot distinguish among the different NP-SLE manifestations (36). From our point of view, only LAC may have a place as a clinically useful biomarker, since its positivity may help in the classification into disease phenotypes and might affect clinical decision making (Table 1). Other molecular biomarkers measured in the serum and in CSF, such as interleukins, chemokines, hormones and complement components, have also been proposed to have a role as diagnostic biomarkers in NP-SLE. Among them we find higher degree of agreement (at least in three different studies) in findings of higher levels of intrathecal IL-6 in NP-SLE patients, especially those with diffuse NP-SLE and acute confusional state (39–45). Although not specific, IL-6 is used in some clinics to assist the diagnosis of such cases and even a role for IL-6 in monitoring of disease activity and response to treatment has been proposed (43, 46). Besides the promising data from mouse studies, studies in humans on the complement cascade have been disappointing so far. The value of INF-α as diagnostic biomarkers in human NP-SLE merits further research (47).

**Table 1:** Strength of evidence for laboratory biomarkers in neuropsychiatric systemic lupus erythematosus in humans.

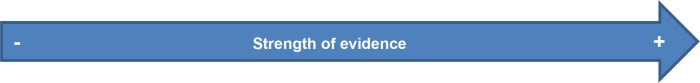		
**EXPERIMENTAL BIOMARKERS**	**VALIDATED BIOMARKERS**	**CLINICALLY USEFUL BIOMARKERS**
- Neurons (anti-neuronal Ab)^s, c^ - Brain reactive autoantibodies (anti-BRAAs)^s^	- Neurons (anti-neuronal Ab)^s^	
- Gangliosides (anti-GA)^s, c^ - Neurofilament (anti-α-internexin)^s, c^ - Microtubule-associated protein 2 (anti-MAP2)^s, c^ - Glial fibrillary acid protein (anti-GFAP)^s, c^ - N-Methyl-D-Aspartate receptor (anti-NMDA, NR2A/B)^s, c^ - Gamma-aminobutyric acid type B receptors (anti-GABA)^s, c^ - Serum lymphocytotoxic antibodies (anti-LCA)^s^ - Triosephosphate isomerase (anti-TPI)^s^ - Brain synaptosomal (anti-BS)^s^ - CNS tissue (anti-CNS)^s^ - Hsp70 (anti-Hsp70-71)^s^ - Alpha-tubulin (anti-α-tubulin)^s^ - Peroxiredoxin (anti-PRDX4)^s^ - Ubiquitin carboxyl-terminal Hydrolase isozyme L1 (anti UCH-L1)^s^ - Splicing factor arginine/serine-rich 3 (anti-SFRS3)^s^ - Brain-derived neurotrophic factor (anti-BDNF)^s^ - Aquoporin 4 (AQP-4)^s^ - Myelin oligodendrocyte glycoprotein (MOG)^s^		
- SSA/Ro (anti-SSA)^s, c^ - Cardiolipin (aCL)^s^	- Lupus anticoagulant^s^ - Ribosomal proteins (anti-ribosomal P)^s^	- Lupus anticoagulant^s^
- Lupus anticoagulant (LAC)^s^ - Ribosomal proteins (anti-ribosomal P)^s, c^ - Sm (anti-Sm)^c^ - U1-RNP (anti-U1-RNP)^c^ - Endothelial cells, Nedd5 (anti-Nedd5)^s^ - Heparan sulfate (anti-HS)^s^ - C1q (anti-C1q)^s^ - Histone H1, H2B, and H3^s^ - S 100 calcium-binding protein B (S100B)^s^ - Neutrophil gelatinase associated lipocalin (NGAL)^s^ - Nitrate nucleosomes (NN)^s^		
- Interleukins: IL-1^s, c^, IL-6^s, c^, IL-8^c^, IL-10^c^, IL-17^c^	- IL-6^c^	- IL-6^c^
- Tumor necrosis factor-alpha (TNF-α) ^s, c^ - Interferon-alpha (IFN-α)^c^ - Interferon-gamma (IFN-γ)^c^ - A proliferation-inducing ligand (APRIL) ^c^ - B-cell activating factor of TNF family (BAFF)^s, c^ - Monocyte chemotactic protein 1 (MCP-1)^c^ - Fractalkine (CX_3_CL1)^c^ - RANTES (CCL5)^c^ - Monokine induced by IFN-γ (MIG)^c^ - Interferon-gamma-inducible 10-kd protein (IP-10)^c^ - Vascular cell adhesion molecule 1 (VCAM-1)^c^ - P-selectin^c^ - Granulocyte colony-stimulating factor (G-CSF)^c^ - α-Klotho^c^ - Kinin system components (Kininogen fractions, kallikreins, and kininase II)^s, c^ - Matrix metalloprotease-9 (MMP-9)^s, c^ - Plasminogen activator inhibitor 1 (PAI-1)^c^ - Prolactin (PRL)^c^ - Soluble terminal complement complex (TCC)^s, c^ - C3 and C4^s, c^ - Fluid phase terminal complement complexes (SC5b-9)^s, c^ - Quotient of alpha2 macroglobulin (Qα2MG)^c^		

*Biomarkers were grouped into three groups: exploratory biomarkers (any biomarker proposed as candidate for diagnosing NP-SLE), validated biomarkers (biomarkers with stronger evidence about an association with NP-SLE, including meta-analysis), and clinically useful biomarkers (biomarkers widely and routinely tested in clinical practice). The type of biomarker is indicated in colors: red for neural cells and constituents, violet for ubiquitous cellular components and green for a miscellaneous group including interleukins, chemokines, hormones, and complement components. c: CSF; s: serum*.

## The Challenges of Establishing a Neuroimaging Biomarker in NP-SLE

Neuroimaging tools provide the ultimate means of obtaining information about the brain. Most neuroimaging tools are low to non-invasive, and are in general subdivided in four categories, employing four different physical principles: reflection and scattering of high frequency sound waves (Ultrasound), mapping the distribution of exogenous molecules in which radioactive atom or atoms are incorporated [Positron Emission Tomography (PET) and Single Photon Emission Computed Tomography (SPECT)], mapping of the attenuation of x-rays through the body (Computer Tomography) and mapping of the radiofrequency signal generated by the nuclei of hydrogen atoms, mostly those incorporated in water (Magnetic Resonance Imaging or MRI). Of these four, two have become the main staple of neuroimaging of disease—MRI and PET. These methods are in many ways complementary to each other, and each provides several means to monitor disease—from gross changes in brain shape and volume, to subtle changes in brain physiology, neurochemistry, and tissue microstructure. These two neuroimaging methods yield clear and unequivocal diagnostic evidence when the source of the damage to the brain is well-delineated and well-defined, as is the case in brain tumors and stroke. Yet, the diagnostic utility of both MRI and PET term “neuroimaging biomarker” in conjunction with brain diseases that involve multiple pathomechanisms that affect the brain in a more subtle way is less obvious. For example, neuroimaging contributes significantly to diagnosis of diseases such as MS (48, 49), Parkinson's disease (50), and Alzheimer's disease (51) but rarely can the diagnosis rely on the findings of neuroimaging alone, and often the neuroimaging results are used in a confirmatory or exclusionary way. Both PET and MRI have been intensely applied to study the brain of SLE and NP-SLE patients, and their role in understanding the disease process and contributing to diagnosis is undisputed. However, the term “neuroimaging biomarker” elicits a degree of sensitivity and most of all specificity that current neuroimaging methods do not provide in NP-SLE.

### Magnetic Resonance Imaging (MRI)

Since its inception in the 1970s, MRI has become the most commonly used neuroimaging tool in diagnosis and research, providing data of high diagnostic value as well as insights into pathological mechanisms underlying these diseases. The astounding versatility of MRI, combined with its non-invasive nature, led to its primary status as the first-stop radiological diagnostic tool, including in SLE patients presenting with neuropsychiatric manifestations. Abnormal brain MRI in SLE and NP-SLE has been reported since the 1980s (52, 53). The most visible aspects of brain pathology available to the radiologist are brain lesions, reflected via hyper/hypointense areas on the images, and gross morphological changes such as global atrophy. Visible changes on MRI are the most immediate tool for a radiological evaluation of brain involvement, and in SLE and NP-SLE these have been regularly used as a standard clinical evaluation tool and as part of NP-SLE diagnosis (54).

#### Conventional MRI and the Clinico-Radiological Paradox in NP-SLE

Despite providing vital evidence about CNS involvement, both morphological changes and brain lesions have not provided a robust link neither to symptoms and disease outcome, nor to the pathological mechanisms underlying NP-SLE. A significant portion (around 40%) of those diagnosed with NP-SLE show no abnormalities on conventional MRI (cMRI), and global measures such as lesion load or brain atrophy do not scale with symptom severity (54–56). The reasons for this apparent failure are not completely understood. Brain tissue damage in NP-SLE is caused by a multitude of pathological processes, the endpoint of which are the visible changes on the MRI. In the most comprehensive prospective study to date that correlated pre-mortem cMRI in NP-SLE with post-mortem histopathology, visible MRI findings reflected global ischemic changes, parenchymal edema, microhemorrhages, gliosis, diffuse neuronal/axonal loss, cerebral infarction, microthromboemboli, and other findings, many of which were attributed to vascular origins (57). This apparent clinico-radiological paradox is the most powerful driving force for establishing neuroimaging biomarkers that not only correlate better with disease outcome, but also provide better insight into the underlying pathology of NP-SLE.

#### Quantitative MRI – Attaching Numbers to Subtle Disease-Modulated Image Changes

In addition to *visible* changes in brain tissue integrity, structure and morphology, MRI can report on *diffuse* changes in brain tissue microstructure, neurochemical composition and physiology. These changes cannot be reported immediately from radiological observations and require additional analysis of the images, typically resulting in a quantitative measure. In quantitative MRI (qMRI), the numeric value assigned to each image unit, or *voxel*, is not the one resulting directly from the image measurement but rather a *calculated value*, typically derived from two or more images. These values can represent intrinsic properties of the MRI signal, or a proxy to a local physiological, neurochemical or a microstructural property of brain tissue. Analyses can be performed based on *regions of interest* (ROI), or on *voxel-by-voxel comparison* following registration of individual quantitative images (or *maps*) to a common brain template. qMRI measures are particularly useful in assessing subtle inter-subject differences in brain regions where there is no apparent damage seen on conventional MRI. In many diseases, these subtle, more diffuse alterations are believed to reflect changes at early stages of the disease (58). Although qMRI can be useful on a single-subject level, most qMRI analyses focus on group differences. Such qMRI methods include relaxometric analyses and magnetization transfer imaging, diffusion weighted and diffusion tensor imaging, perfusion based imaging, and magnetic resonance spectroscopy. See Supplementary Table 2 for a brief description of quantitative MRI methods.

**Figure 2:**
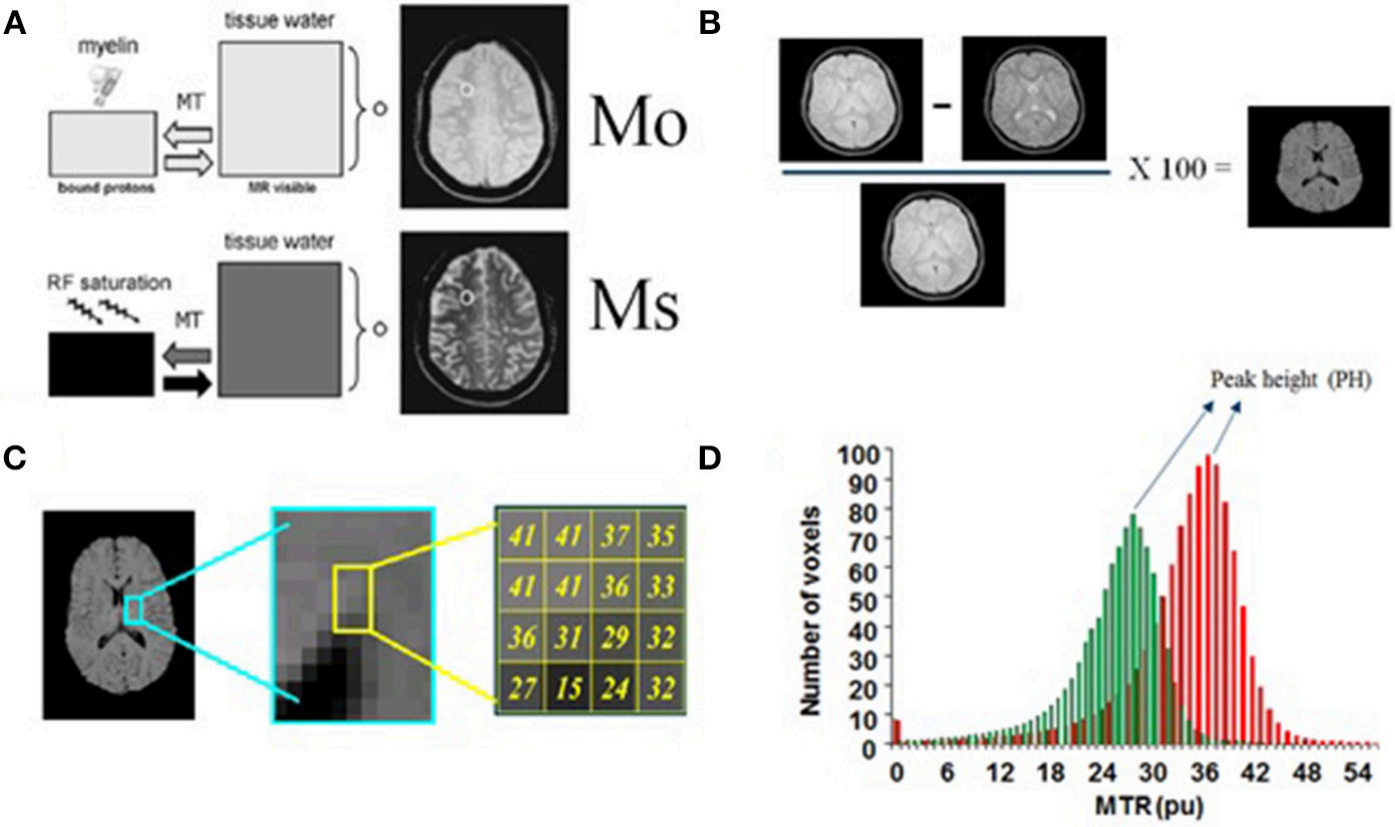
Basis of Magnetization Transfer Imaging. **(A)** This technique is based on the application of off-resonance radiofrequency pulses. M_0_: proton density image or intensity of voxels without saturation, M_s_: Bound protons or intensity of voxels saturated. **(B)** Measurement of signal intensity with and without the application of these pulses allows the calculation of an index called the magnetization transfer ratio (MTR) which is defined as (M_0_-M_s_/M_0_) × 100%. **(C)** MTR histogram: this technique takes a ratio of the two images on a voxel-by-voxel basis (brain pixels). **(D)** The histogram peak height (HPH), a MTR histogram-derived measure, accounts for the proportion of brain pixels at the most common MTR value. **(A)** partially adapted from Grossman et al. (61).

qMRI has been used in the study of NP-SLE and in earnest attempt to provide quantitative measures for the effects of NP-SLE on the brain:

- *MR Relaxometry*: Early studies looked at the usefulness of an automated evaluation of mean cortical T_2_ values as a possible marker for cortical changes in NP-SLE (59). The study acknowledged the potential confound of increased CSF fraction due to atrophy, which was more prominent in the NP-SLE population. T_2_ relaxometry was later incorporated in a multisequence study of NP-SLE in a small group of patients, in which MTR, MRS and DWI were also recorded (60). This is the first study in which several qMRI methods were applied, and whole brain values of the various modalities were correlated with each other. Several significant correlations were detected among modalities, but the study did not report on correlations with clinical parameters, thus the relationship of the quantitative MRI measures with any pathomechanism in NP-SLE is not explored.
- *Magnetization transfer imaging (MTI) and magnetization transfer ratio (MTR)*: Several studies have highlighted the usefulness of MTR, an MTI-derived parameter, as a potential marker for brain microstructural changes in NP-SLE. Most studies focused on analysis of whole brain or tissue-specific (gray matter, white matter) histograms of MTR values (Figure 2) (61). Histograms of qMRI values in neurological disorders, development and aging are commonly applied (62–68). They provide a cumulative estimation of a quantitative measure, and thus lack any spatial information. They are, however, sensitive to diffuse, global effects, and successive studies have shown the sensitivity of MTR histograms to a variety of disease related clinical factors (69–73), including to the presence of specific antibodies. It was recently shown that white matter MTR histogram peak heights (HPH) were significantly lower in inflammatory NP-SLE patients than in NP-SLE patients diagnosed as ischemic, as well as than SLE patients with no NP complaints and healthy controls (74). The mechanism by which the MTR is decreased in NP-SLE is not clear, but its reversibility upon successful treatment suggests intracellular edema and gliosis as the associated pathomechanisms (75).
- *Diffusion weighted imaging (DWI) and diffusion tensor imaging (DTI):*Abnormal water diffusivity in brain white matter in NP-SLE has been reported in several studies (2, 76–80) and a review that summarizes many of these findings has been recently published (81). Global and local microstructural abnormalities, reflected in increase in mean diffusivity (MD) and decrease in fractional anisotropy (FA), were reported in most of the studies selected in this review. As in most MTI studies, most DWI and DTI studies did not explicitly exclude “visible” white matter hyperintensities from the analysis, albeit some of the studies made the point that the data suggests microstructural differences in NP-SLE in normal appearing white matter (82). Of importance is to note that several studies reported DTI abnormalities in SLE patients with no NP symptoms (non-NP-SLE). The review mentioned above dedicates a paragraph to DTI findings in non-NP-SLE compared to healthy controls, citing several studies that report positive findings, such as reduced FA in frontal white matter (78) and increased MD and reduced FA in the corpus callosum (83).
- *Proton Magnetic resonance spectroscopy (1H-MRS):* This technique is a non-invasive test that permits chemically specific, non-invasive measurements of the concentration of neuronal metabolites. In the brain there are about 20 such metabolites on which MRS can reliably report. Some have known functions such as neurotransmitters (glutamate, GABA), some are involved in energy metabolism [Lactate, creatine (tCr)] and some are uniquely (or preferentially) located in specific cell types (N-acetylasparate (NAA) in neurons, myo-inositol (MI) in astrocytes) (84, 85). Concentrations of metabolites are sometime modulated by disease, as well as their ability to freely move (or diffuse) in the cytoplasm. MRS measurements are either performed on a single volume of interest (VOI) positioned on an area of interest, or in multivoxel mode (spectroscopic imaging). This technique has been used in SLE studies where differences in the concentrations of several metabolites (relative to tCr) have been reported (86). Lower NAA and higher Cho and MI levels have been reported in SLE and NP-SLE patients when compared to healthy controls, suggesting decreased neuronal function and glial activation, respectively (73, 80, 87–90). More recently, lower NAA changes in NP-SLE patients when compared with SLE and in SLE with high disease activity when compared with low activity were found (91, 92). Diffusion weighted MRS (DWS) probes the mobility of intracellular metabolites in the cytoplasm, and is thus able to detect cytomorphological changes in disease (93). One such study reported increased diffusivity of glial metabolites in NP-SLE compared to healthy controls, with no concomitant change in neuronal metabolites, suggesting glial involvement (94).
- *Perfusion weighted MRI:* Despite the strong vascular aspect of NP-SLE, perfusion-based neuroimaging methods do not provide unequivocal information regarding NP-SLE-related modulation of cerebral blood flow and cerebral blood volume. Some studies report global and regional increase in cerebral blood flow (CBF) in NP-SLE as measured by MRI (95, 96). A recent study reported decreases in CBF in a well-defined white matter region, the centrum semiovale, with good sensitivity and specificity. The systematic attribution of the NP symptoms to SLE in this study was particularly tight and the high statistical significance of the results show big promise (97).
- *Functional MRI* (fMRI): Several studies investigated functional deficits in SLE and NP-SLE, and comprehensive review that summarizes the findings from these studies was published in 2016 (98). Task-related studies spanned a variety of tasks aimed at investigating working memory (99), sensory integration (100), and emotional responses (101). Resting state fMRI studies focused on functional networks that can be linked to cognitive and behavioral deficits (102, 103). Findings of hypo- and hyperactivation linked to symptoms and disease state were reported, and overall shed light on potential links between NP symptoms and brain functional deficits on local and network levels. Despite the usefulness of these studies, the possibility of generating a neuroimaging biomarker specific to NP-SLE based on brain function is not particularly promising, given the heterogeneity of the underlying pathomechanisms of NP-SLE and the wide range of NP symptoms.

### Positron Emission Tomography (PET) and Single Photon Emission Computed Tomography (SPECT)

Positron Emission Tomography is widely regarded as a highly useful neuroimaging tool in the clinic, in particular in oncology, where radioligands with high specificity to tumors have been developed and can provide early vital information on the presence of tumors in early stages of the disease (104). In addition, PET provides also useful information on physiological as well as on tissue microstructural and composition changes in neurological disorders, either through ligands that bind to abnormal formations of proteins involved in diseases such as Alzheimer's disease (105) or via reporting on abnormal metabolism, sometime associated with early signs of neurodegeneration and inflammation (106). Positron Emission Tomography studies in SLE and NP-SLE showed potential usefulness, in particular studies with ^18^F-FDG (fluorodeoxyglucose) showed increased metabolism in response to neuroinflammation and correlated with inflammatory status also under follow up (107). Positron Emission Tomography radioligands that bind to translocator protein (TSPO) are currently being investigated as potential markers for microglial activation in response to inflammation (108) and may provide an additional probe for neuroinflammation in NP-SLE (109).

Single photon emission computed tomography (SPECT) has also been used in SLE patients. Some SPECT studies report decreases in blood flow in NP-SLE in watershed regions (110). Glucose uptake, as detected by PET using 18-FDG, is intimately linked to blood flow, as they are both modulated by cellular metabolism (111). Several 18-FDG PET studies reported regional hypometabolism in NP-SLE (107, 112), but others provide evidence also for *increase* in glucose metabolism in white matter regions, consistent with inflammatory response (96, 113). Taken together, it appears that hypo- and hypermetabolism may relate to two different yet coexisting aspects of brain involvement in SLE.

## Biomarkers in NP-SLE: Future Perspectives

As previously shown, yet despite years of efforts of the NP-SLE scientific community, the number of clinically useful biomarkers and even of validated biomarkers is embarrassingly modest. From our point of view a series of scientific challenges in the field have yet to be overcome:

- The *NP-SLE definition* is a challenge by itself. Since 1999, research in this field has been guided by the ACR case definitions for NP-SLE syndromes including a group of 19 complex and uncommon neuropsychiatric manifestations involving both the central (12 syndromes) and peripheral (7 syndromes) nervous system (114). Researchers have mainly focused on analyzing biomarkers in NP-SLE defined as a group based on these definitions without taking into account the underlying pathophysiological mechanism. Using such heterogeneous manifestations as a group may be problematic since it may include manifestations with obviously different underlying pathophysiological mechanisms, e.g., stroke and acute confusional state. Clinicians and researchers in the field would benefit from resolving the problem of heterogeneity by using biomarkers capturing the different aspects of nervous involvement in SLE. Borowoy et al. demonstrated how autoantibody associations depend on the NP-SLE definition used (115). In clinical practice, the gold standard is a diagnosis conducted by a multidisciplinary expert clinical team. Furthermore, the diagnosis in NP-SLE is made phenotypically according to the suspected underlying pathophysiological mechanism (inflammatory, thrombotic or infrequently coexistence of both) which is critical for guiding treatment. Phenotypic characterization is important in clinical practice but may be also in research. A given phenotype may arise from a diverse set of biochemical processes and its changes in the brain may be captured by a diverse set of neuroimaging techniques. The identification of a biochemical and neuroimaging subset of factors that underlie a specific phenotype or certain NP-SLE manifestation should be preferable in future research and more applicable in clinical practice.
- Apart from the heterogeneity of the groups, the *small sample size due to the low prevalence* is one of the common denominators of studies describing new potential biomarkers in NP-SLE. Given the rarity and complexity of NP-SLE, collaborative efforts, using pooled clinical, laboratory, and neuroimaging data sets are needed. Much larger studies will allow for more specific hypothesis about for example a specific phenotype or NP-SLE manifestation, permit the use of biomarker combinations and analyze the relations among them. Furthermore, collaboration will facilitate performing the first serious trial with well-known drugs or even additional therapeutic choices, giving the opportunity to assess the role of these biomarkers in monitoring of disease activity and response to treatment.
- *Study design*: A reason for the minimal clinical impact of reported biomarkers may be that most of these studies report differences between NP-SLE patients and SLE at a group level while physicians have to make clinical decisions individually. Furthermore, in clinical practice, when a SLE patient presents with NP complaints it is obligatory first to exclude other potential causes before these symptoms are attributed to SLE. Most of the studies compare the higher presence of a certain biomarker in SLE patients with and without NP-SLE manifestations, remaining uncertain if this biomarker profiles are unique to NP-SLE or may be present in other mimicking neuropsychiatric disorders; only a few studies have used a group of patients with other neuropsychiatric disease (e.g., MS or septic meningitis) as control groups (116). For example, B-cell activating factor of TNF family or matrix metalloprotease-9 have been proposed as exploratory biomarker in NP-SLE because its higher positivity when compared with SLE; however both biomarkers have also been proposed as biomarkers in patients with MS (117).

In the particular case of ***laboratory biomarkers***, the next promising aspects in NP-SLE should be addressed in the near future.

- *Identification of new neuronal surface antigens* which are responsible for NP-SLE. The antigen identification paradigm which has been successfully used with limbic encephalitis may be applied on NP-SLE to recognize unknown neuronal cell-surface protein(s) (34). Determination of neuronal immunoreactivity in different areas of brain and cerebellum of homogeneous clinical and radiological NP-SLE groups may be analyzed and afterwards correlated with clinical symptoms and MRI characteristics. To identify the target antigen cultured neurons and mass spectrometry could be used. Lastly, brains of knock out animal models or cells deprived of the suspect antigen by siRNA knock down may confirm the specificity of these candidate autoantibodies (118).
- *Omics*: In the last years, laboratory biomarker discovery has benefit from the development of omics technologies such as genomics or immune-proteomics, which has successfully increased the list of exploratory biomarkers in many diseases (119). These techniques give the opportunity to explore a wide spectrum of biomarkers in a more comprehensive and unbiased way. Autoantigen microarrays have already been used in NP-SLE (120, 121). For example, van der Meulen *et al* have shown how a profile of IgG and IgM autoantibodies against 15 antigens may help to differentiate NP-SLE from non-NP-SLE (120). The potential for false positive discoveries using these techniques is high; reproduction of this data and selection of best candidates may be a next step before validation in large-scale independent cohorts (122).
- *Complement cascade and IFN-alpha*: The exciting area of research in NP-SLE mice models on complement cascade and IFN-alpha needs to be translated to human NP-SLE. The study of these two biomarkers may lead to a better understanding of pathogenic underlying mechanisms of synapse loss and will probably open the door to the use of new therapeutic strategies in NP-SLE (e.g., Eculizumab, a humanized monoclonal antibody blocking the generation of terminal complement components C5a and C5b-9, and Sifalimumab, a human anti-IFN-a monoclonal antibody).
- *Understanding of the BBB* in NP-SLE. Brain tissue-reactive antibodies in NP-SLE are thought to be synthesized in the CNS, but also in peripheral organs (lymph nodes and bone marrow). In the last case it was proposed that these autoantibodies must pass through pathologically permeable BBB to exert an effect upon neurons. Although an important role of BBB has been supposed, we need better understand the role of BBB in human NP-SLE. A recent study in mice questions the widely accepted hypothesis of a disrupted BBB and suggests a dysfunction of the blood-cerebrospinal fluid barrier in the choroid plexus underlying brain exposure to these neuropathic antibodies (123). More studies in humans comparing serum and CSF and using quotients are warranted.

Future research on ***neuroimaging biomarkers*** for NP-SLE will need to address the next factors

**Figure 3:**
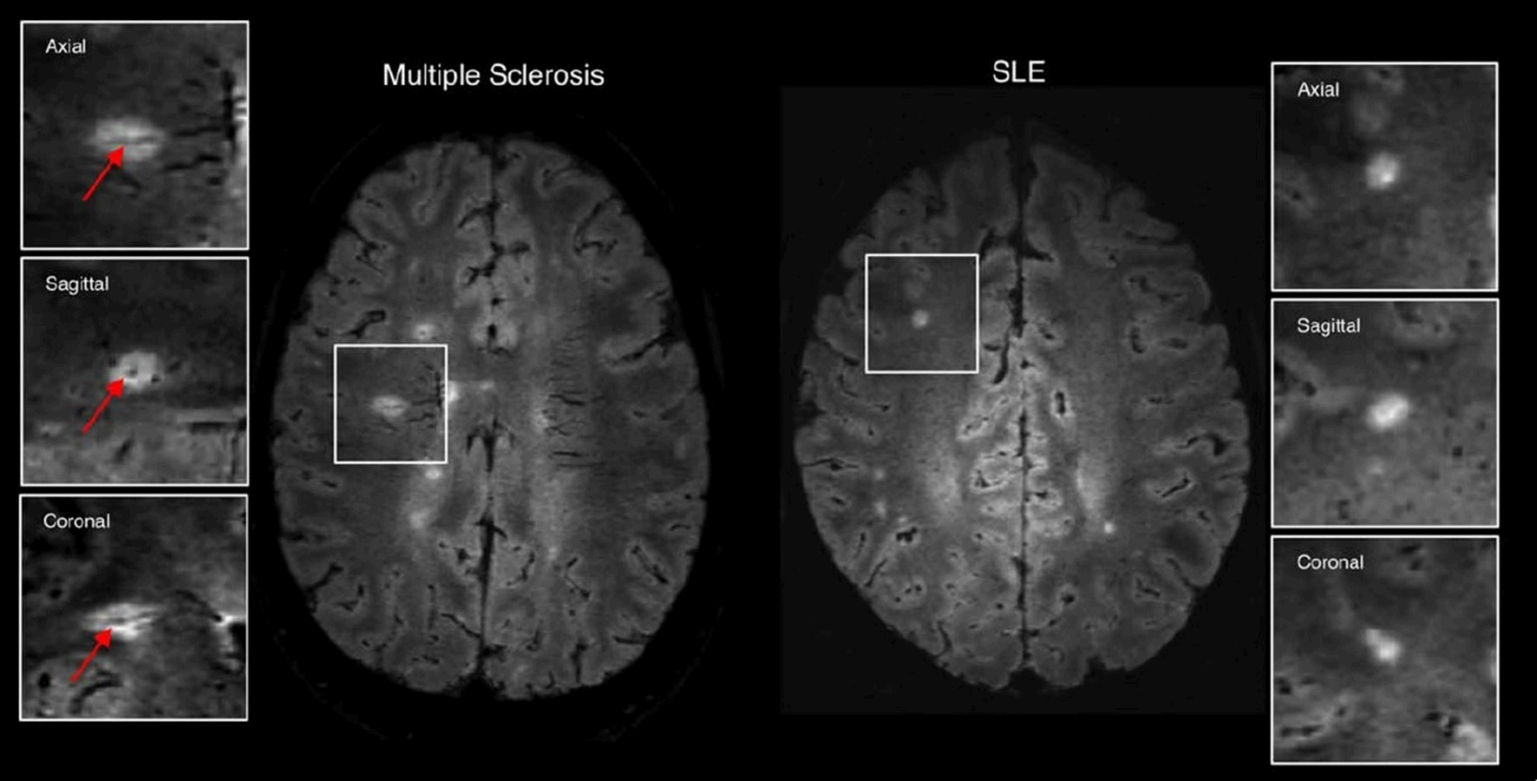
Examples of 3T FLAIR* imaging demonstrating the central vessel sign (arrows) in multiple sclerosis **(left)** but not in systemic lupus erythematosus **(right)**. Figure adapted with permission from Maggi et al. (119).

- *Another look at cMRI: the case for more sophisticated characterization of lesions*: In recent years, characterization of lesions in neurological disorders has advanced far beyond the basic lesion count or lesion load. Most notable is the work related to lesions and their pathological classification in MS. It has long been postulated that lesion location and not lesion load as a robust surrogate marker of neuropsychological impairment in patients with MS (124). Link between lesion location and cognitive function was shown to be significant in some studies (125, 126). The latest, and most significant step in characterization of white matter lesions in MS came after examining the spatial relationship between lesions and large veins in a visualization method that superimposes FLAIR images containing lesion spatial information, and ${\text{T}}_{2}^{*}$-weighted images, showing vein distribution in great detail (Figure 3) (127). It was found that concentric co-localization of a white matter lesion with a vein that passes through it, termed central vein sign (CVS), is highly specific to the early stages of MS and has been swiftly adopted as a biomarker mandated for MS diagnosis by the North American Imaging in Multiple Sclerosis Cooperative (NAIMS). The presence and development of CVS are well-explained by a neuroinflammatory mechanism with a vascular origin, and CVS has been shown to differentiate well-between MS and other central nervous system inflammatory vasculopathies including SLE (128), although not without cautionary remarks (129).
- *Same picture – multiple views: the role of multimodal neuroimaging in NP-SLE*: The multifactorial nature of NP-SLE, combined with the lack of specificity of most imaging modalities to any particular pathomechanism makes the quest for a “silver bullet” diagnostic tool unrealistic. Even PET ligands for TSPO, initially assumed to have high specificity to microglial activation and thus to inflammation, have been shown to be less specific than the initial expectations A natural approach is to combine several neuroimaging markers, each highlighting a different aspect of the disease in an approach that uses a multivariate analysis in one way or the other. This approach has been suggested for other neuropsychiatric and neurological disorders, especially for those with little overt brain damage and complex underlying mechanisms such as major psychiatric disorders. In NP-SLE, most of the clinical MRI protocols are intrinsically multimodal, and in many sites additional imaging data that is not directly clinical are acquired. Several approaches for multimodal data analysis of neuroimaging data have been proposed, aimed primarily at developmental psychiatric and neurodegenerative disorders but also to stroke and tumors (130–136).

The application of the previous laboratory and radiological techniques in NP-SLE will produce hundreds of exploratory biomarkers where complicated statistical methodology is required. Analytical methods such as supervised *machine learning* (ML) promise help solving this problem and advance the development of biomarkers in the near future. This technique uses algorithms to automatically extract information from data that can be applied at the individual level to make predictions therefore with a higher level of clinical translation. Furthermore, ML can be applied to laboratory biomarkers but also to neuroimaging data, since these methods are sensitive to spatially distributed and subtle effects (137). For example, neuroimaging data are intrinsically large, if it is considered that the number of pixels in MRI data sets is in the order of 1 × 10^7^ (ten million) pixels, and several such sets are combined together to yield the multimodal data set.

## Conclusion

Besides the enormous progress made in the area, specific laboratory and neuroimaging biomarkers in NP-SLE are scarce and their validation as useful biomarkers in routine clinical practice is far from becoming reality. In the present paper, we have proposed several research paths that may help to overcome some of the obstacles that hamper the validation of laboratory and neuroimaging features useful as diagnostic, prognostic or response to treatment biomarkers. We are convinced that many of these obstacles will only be overcome thanks to large collaborative efforts. Importantly, the difficulties in establishing a single imaging or laboratory biomarker for NP-SLE point toward a concerted interdisciplinary effort to establish an optimal set of imaging and laboratory markers that will correlate best with disease phenotype, progression and severity.

## Author Contributions

CM-C, GS-B, TH, MB, and IR have made significant contributions to the design, the literature research, and the writing of the manuscript. All authors reviewed and approved the final version of the manuscript.

### Conflict of Interest Statement

The authors declare that the research was conducted in the absence of any commercial or financial relationships that could be construed as a potential conflict of interest.
