# Supplementary material for: Laboratory and Neuroimaging Biomarkers in Neuropsychiatric Systemic Lupus Erythematosus: Where Do We Stand, Where To Go?
Source: Front Med (Lausanne). 2018 Dec 4;5:340. doi: 10.3389/fmed.2018.00340 (PMC6288259; doi:10.3389/fmed.2018.00340)
Supplement: Supplementary file 1 [file Data_Sheet_1.docx]

| **Supplementary Table 1. Autoantibodies related with human NPSLE** | | | | |
| --- | --- | --- | --- | --- |
| **Specific target** | **Tested in** | **Mechanism/Function** | **Association** | **Reference** |
| ***Neuronal cells and constituents*** | | | | |
| Neurons (anti-neuronal Ab) | Serum/CSF | Reaction to neuronal components | Lupus psychosis and diffuse NP-SLE. May correlate with CNS activity | (1-10) |
| Brain reactive autoantibodies (anti-BRAAs) | Serum | Antibodies binding integral membrane proteins of the brain | Psychosis and/or seizures | (11, 12) |
| Gangliosides (AGA) | Serum/CSF | Leakage of the BBB and bind to neuronal gangliosides to create a neuromuscular block | Cognitive dysfunction, depression, peripheral neuropathy and headache | (13-18) |
| Neurofilament (anti-alpha internexin) | Serum/CSF | Cortical and hippocampal neuron apoptosis | NPSLE | (19, 20) |
| Microtubule-associated protein 2 (anti-MAP2) | Serum/CSF | MAP2 is important in the control of cytoskeletal integrity and neuronal functions | Diffuse manifestations | (21-23) |
| Glial fibrillary acid protein (anti-GFAP) | Serum | Antibodies directed against glial cell-related antigens | Organic/major type | (24-26) |
| N-Methyl-D-Aspartate receptor (Anti-NMDA, Anti-NR2A/B) | Serum/CSF | Autoantibody against receptor/Hippocampus and amygdala atrophy. Apoptosis | Diffuse NPSLE. No association with NP syndromes. | (27-31) |
| Gamma-aminobutyric acid type B receptors (Anti-GABA) | Serum/CSF | Autoantibodies have a disruptive effect on GABA-mediated immune response | NPSLE | (32) |
| Serum lymphocytotoxic antibodies (Anti-LCA) | Serum | Activity against neurons in the cortex, cerebellum, and caudate nucleus | NPSLE. Cognitive impairment. | (33-35) |
| Triosephosphate isomerase (Anti-TPI) | Serum/CSF | Microtubule stabilization | NPSLE. Higher frequency aseptic meningitis | (23, 36) |
| Brain synaptosomal (anti-BS) | Serum | Antibodies binding components of synaptosoma (not well defined) | NPSLE | (37) |
| CNS tissue (Anti-CNS) | Serum | Organ-specific Abs against epitopes of CNS tissue | Cerebral involvement in connective tissue diseases | (18) |
| Hsp70 (Anti-Hsp70-71) | Serum | Protect brain against ischemia | NPSLE | (23) |
| Alpha-tubulin | Serum | The main component of microtubules | NPSLE | (38) |
| Peroxiredoxin (Anti-PRDX4) | Serum | Protect living cells from oxidative damages. Involved in cell proliferation and differentiation | NPSLE | (39) |
| Ubiquitin carboxyl-terminal Hydrolase isozyme L1 (Anti UCH-L1) | Serum | Involved in neuronal differentiation, synaptic functions, and contextual memory | NPSLE | (39) |
| Splicing factor  arginine/serine-rich 3 (Anti-SFRS3) | Serum | Involved in regulation of alternative splicing of precursor mRNA including its own mRNA | NPSLE | (39) |
| Brain-derived neurotrophic factor (BDNF) | Serum | Support the survival of neurons | Psychiatric symptoms SLE | (40, 41) |
| Aquoporine 4 (AQP-4) | Serum | Complement- and cell-mediated astrocyte cytotoxicity, leads to inflammatory response with oligodendrocyte injury and demyelination | Neuromyelitis optica spectrum disorder (NMOSD)/SLE overlap | (42) |
| Myelin oligodendrocyte glycoprotein (MOG) | Serum | Inflammation and myelin destruction without astrocyte injury | NPSLE | (43) |
| ***Ubiquitous cellular components*** | | | | |
| SSA/Ro | Serum/CSF | Unknown | Psychosis, chorea, cognitive dysfunction. NP damage. | (44, 45) |
| Cardiolipin | Serum/CSF | Thrombotic occlusion, atherosclerosis, endothelial activation, mild inflammation, inhibitory effect on brain cells | Focal NP-SLE. Stroke, seizure and cognitive impairment. | (10) |
| Lupus anticoagulant (LAC) | Serum | Thrombotic occlusion | Focal/Ischemic NP-SLE. Intracranial thrombosis | (10, 46) |
| Ribosomal proteins | Serum/CSF | Cross-react with a neuronal surface protein to initiate calcium influx and apoptosis | NPSLE. Predictor of lupus psychosis. | (10, 46, 47) |
| Sm | CSF | Neural toxicity | NPSLE. Acute confusional state. Seizure, psychiatric disorders, peripheral neuropathy | (48) |
| U1-RNP | CSF | May act as an inducer of pro-inflammatory cytokines. | NPSLE. Psychiatric disorders, peripheral neuropathy | (48, 49) |
| Endothelial cells, Nedd5 | Serum | Nedd5 relocated from cytoplasm to the plasma membrane of EAhy926 endothelial cells after apoptotic stimuli. Endothelial dysfunction | Psychiatric manifestations (Psychosis, mood disorders). Ischemic stroke | (50-52) |
| Histone H1, H2B and H3 | Serum | Immune complexes | NPSLE | (53, 54) |
| S 100 calcium-binding protein B (S100B) | Serum | Marker for brain damage and blood-brain barrier disruption | NPSLE (ACS, seizures, CVA and psychosis). Cognitive impairment in children with SLE | (55-58) |
| Neutrophil gelatinase associated lipocalin (NGAL) | Serum | Neuroinflammatory mediator | NPSLE. Cognitive dysfunction in children with SLE | (59) |
| Nitrate nucleosomes | Serum |  | NPSLE | (60) |
| Anti-Heparan sulfate | Serum |  | NPSLE | (54) |
| Fractalkine (CX_3_CL1) | Serum/CSF | Chemokine, type 1 transmembrane protein synthesized by endothelial cells, observed in CNS | NPSLE | (61) |
| C1q | Serum |  | Diffuse NPSLE | (62) |
| ***Interleukines*** | | | | |
| IL-1 | CSF/serum |  | NPSLE. Inflammatory NPSLE, demyelination. | (63, 64) |
| IL-6 | CSF/serum |  | NPSLE. Lupus psychosis. Inflammatory NPSLE, demyelination. Multiple ischemic foci. | (63-73) |
| IL-8 | CSF |  | NPSLE | (63, 65-67, 70) |
| IL-10 | CSF and serum |  | No association | (67, 71) |
| IL-17 | CSF |  | NPSLE | (66) |
| Tumor necrosis factor-alpha (TNF-a) | Serum/CSF |  | NPSLE | (63, 65) |
| Interferon-alpha (IFN-a) | CSF |  | NPSLE. Lupus psychosis | (71, 74, 75) |
| Interferon-gamma (IFN-γ) | CSF/Serum |  | NPSLE, multiple ischemic foci | (64) |
| A proliferation-inducing ligand (APRIL) | CSF |  | NPSLE. Relation with fatigue. | (68, 76) |
| B-cell activating factor of TNF family (BAFF) | Serum/CSF |  | NPSLE | (68, 76) |
| ***Chemokines*** | | | | |
| Monocyte chemotactic protein 1 (MCP-1) | CSF |  | NPSLE | (65-67, 77) |
| RANTES | CSF |  | NPSLE | (65, 66) |
| Monokine induced by IFN-γ (MIG) | CSF |  | NPSLE | (65) |
| Interferon-gamma-inducible 10-kd protein (IP-10) | CSF |  | NPSLE | (65, 67) |
| VCAM-1 | CSF |  | NPSLE | (66) |
| P-selectin | CSF |  | NPSLE | (66) |
| G-CSF | CSF |  | NPSLE | (67) |
| α-Klotho | CSF | Single-pass transmembrane protein | NPSLE | (78) |
| Kinin system components (Kininogen fractions, kallikreins, and kininase II) | Serum/CSF |  | NPSLE | (63) |
| Matrix metalloprotease-9 (MMP-9) | Serum/CSF |  | NPSLE. Association with small-vessel cerebral vasculopathy en increased rist of cerebral ischemic events | (79, 80) |
| ***Fibrinolytic factors*** | | | | |
| Plasminogen activator inhibitor 1(PAI-1) | CSF |  | NPSLE | (81) |
| ***Hormones*** | | | | |
| Prolactin (PRL) | CSF |  | NPSLE | (69) |
| ***Complement components*** | | | | |
| Soluble terminal complement complex (TCC) | Serum/CSF |  | No association | (71) |
| C3 and C4 | Serum/CSF |  | C3 low in serum and high in CSF related to inflammatory NPSLE. Low C4 and ischemic NPSLE. | (62, 72, 82, 83) |
| Fluid phase terminal complement complexes (SC5b-9) | Serum/CSF |  | NP-SLE | (84) |
| ***Quotient*** | | | | |
| Quotient of alpha2 macroglobulin (Qα2MG) | CSF | Evaluation blood-brain barrier integrity | BBB integrity (NPSLE) | (72) |
| CNS: central nervous system;CSF: cerebrospinal fluid; NPSLE: Neuropsychiatric systemic lupus erythematosus; | | | | |

**REFERENCES**

1. How A, Dent PB, Liao SK, Denburg JA. Antineuronal antibodies in neuropsychiatric systemic lupus erythematosus. Arthritis Rheum. (1985) 28:789-95.

2. Denburg JA, Carbotte RM, Denburg SD. Neuronal antibodies and cognitive function in systemic lupus erythematosus. Neurology. (1987). 37:464-7.

3. Avinoach I, Amital-Teplizki H, Kuperman O, Isenberg DA, Shoenfeld Y. Characteristics of antineuronal antibodies in systemic lupus erythematosus patients with and without central nervous system involvement: the role of mycobacterial cross-reacting antigens. Isr J Med Sci. (1990) 26:367-73.

4. Hanly JG, Walsh NM, Fisk JD, Eastwood B, Hong C, Sherwood G, et al. Cognitive impairment and autoantibodies in systemic lupus erythematosus. Br J Rheumatol. (1993) 32:291-6.

5. Tishler M, Alosachie I, Chapman Y, Korcyn A, Lorber M, Mevorach D, et al. Anti-neuronal antibodies in antiphospholipid syndrome with central nervous system involvement: the difference from systemic lupus erythematosus. Lupus. (1995) 4:145-7.

6. Isshi K, Hirohata S. Differential roles of the anti-ribosomal P antibody and antineuronal antibody in the pathogenesis of central nervous system involvement in systemic lupus erythematosus. Arthritis Rheum. (1998) 41:1819-27.

7. Zhang X, Shu H, Zhang F, Tian X, Dong Y. Cell-ELISA detection of antineuronal antibodies in central nervous system involvement in systemic lupus erythematosus. Ann Rheum Dis. (2007) 66:530-2.

8. Kang EH, Shen GQ, Morris R, Metzger A, Lee EY, Lee YJ, et al. Flow cytometric assessment of anti-neuronal antibodies in central nervous system involvement of systemic lupus erythematosus and other autoimmune diseases. Lupus. (2008) 17:21-5.

9. Cojocaru IM, Cojocaru M, Botnaru L, Miu G, Sapira V, Tanasescu R. Detection of serum of IgG anti-neuronal antibodies in systemic lupus erythematosus patients with central nervous system manifestations. Rom J Intern Med. (2010) 48:267-9.

10. Ho RC, Thiaghu C, Ong H, Lu Y, Ho CS, Tam WW, et al. A meta-analysis of serum and cerebrospinal fluid autoantibodies in neuropsychiatric systemic lupus erythematosus. Autoimmun Rev. (2016) 15:124-38.

11. Tin SK, Xu Q, Thumboo J, Lee LY, Tse C, Fong KY. Novel brain reactive autoantibodies: prevalence in systemic lupus erythematosus and association with psychoses and seizures. J Neuroimmunol. (2005) 169:153-60.

12. Hanly JG, Fisk JD, Eastwood B. Brain reactive autoantibodies and cognitive impairment in systemic lupus erythematosus. Lupus. (1994) 3:193-9.

13. Hirano T, Miyajima H, Taniguchi O, Ueda A, Takai S, Hashimoto H, et al. Anti asialo GM1 antibody detected in the patients' sera from systemic lupus erythematosus and Behcet's diseases with neurological manifestations. Jpn J Med. (1988) 27:167-71.

14. Chen Y, Wu F, Hou L, Ma S, He X. Antiganglioside antibodies in cerebrospinal fluid of children with neuropsychiatric lupus erythematosus. Chin Med J (Engl). (1997) 110:594-7.

15. Labrador-Horrillo M, Martinez-Valle F, Gallardo E, Rojas-Garcia R, Ordi-Ros J, Vilardell M. Anti-ganglioside antibodies in patients with systemic lupus erythematosus and neurological manifestations. Lupus. (2012) 21:611-5.

16. Pereira RM, Yoshinari NH, De Oliveira RM, Cossermelli W. Antiganglioside antibodies in patients with neuropsychiatric systemic lupus erythematosus. Lupus. (1992) 1:175-9.

17. Galeazzi M, Annunziata P, Sebastiani GD, Bellisai F, Campanella V, Ferrara GB, et al. Anti-ganglioside antibodies in a large cohort of European patients with systemic lupus erythematosus: clinical, serological, and HLA class II gene associations. European Concerted Action on the Immunogenetics of SLE. J Rheumatol. (2000) 27:135-41.

18. Weiner SM, Klein R, Berg PA. A longitudinal study of autoantibodies against central nervous system tissue and gangliosides in connective tissue diseases. Rheumatol Int. (2000) 19:83-8.

19. Robbins ML, Kornguth SE, Bell CL, Kalinke T, England D, Turski P, et al. Antineurofilament antibody evaluation in neuropsychiatric systemic lupus erythematosus. Combination with anticardiolipin antibody assay and magnetic resonance imaging. Arthritis Rheum. (1988) 31:623-31.

20. Lu XY, Chen XX, Huang LD, Zhu CQ, Gu YY, Ye S. Anti-alpha-internexin autoantibody from neuropsychiatric lupus induce cognitive damage via inhibiting axonal elongation and promote neuron apoptosis. PloS One. (2010) 5:e11124.

21. Williams RC, Jr., Sugiura K, Tan EM. Antibodies to microtubule-associated protein 2 in patients with neuropsychiatric systemic lupus erythematosus. Arthritis Rheum. (2004) 50:1239-47.

22. Yamada Y, Nozawa K, Nakano S, Mitsuo Y, Hiruma K, Doe K, et al. Antibodies to microtubule-associated protein-2 in the cerebrospinal fluid are a useful diagnostic biomarker for neuropsychiatric systemic lupus erythematosus. Mod Rheumatol. (2016) 26:562-8.

23. Lefranc D, Launay D, Dubucquoi S, de Seze J, Dussart P, Vermersch M, et al. Characterization of discriminant human brain antigenic targets in neuropsychiatric systemic lupus erythematosus using an immunoproteomic approach. Arthritis Rheum. (2007) 56:3420-32.

24. Alessandri C, Conti F, Valesini G. Role of anti-glial fibrillary acidic protein antibodies in the pathogenesis of neuropsychiatric systemic lupus erythematosus should be clarified: comment on the article by Trysberg et al. Arthritis Rheum. (2004) 50:1698-9.

25. Trysberg E, Nylen K, Rosengren LE, Tarkowski A. Neuronal and astrocytic damage in systemic lupus erythematosus patients with central nervous system involvement. Arthritis Rheum. (2003) 48:2881-7.

26. Sanna G, Piga M, Terryberry JW, Peltz MT, Giagheddu S, Satta L, et al. Central nervous system involvement in systemic lupus erythematosus: cerebral imaging and serological profile in patients with and without overt neuropsychiatric manifestations. Lupus. (2000) 9:573-83.

27. Tay SH, Fairhurst AM, Mak A. Clinical utility of circulating anti-N-methyl-d-aspartate receptor subunits NR2A/B antibody for the diagnosis of neuropsychiatric syndromes in systemic lupus erythematosus and Sjogren's syndrome: An updated meta-analysis. Autoimmun Rev. (2017) 16:114-22.

28. Lauvsnes MB, Omdal R. Systemic lupus erythematosus, the brain, and anti-NR2 antibodies. J Neurol. (2012) 259:622-9.

29. Hirohata S, Arinuma Y, Yanagida T, Yoshio T. Blood-brain barrier damages and intrathecal synthesis of anti-N-methyl-D-aspartate receptor NR2 antibodies in diffuse psychiatric/neuropsychological syndromes in systemic lupus erythematosus. Arthritis Res Ther. (2014) 16:R77.

30. Steup-Beekman G, Steens S, van Buchem M, Huizinga T. Anti-NMDA receptor autoantibodies in patients with systemic lupus erythematosus and their first-degree relatives. Lupus. (2007) 16:329-34.

31. Hanly JG, Robichaud J, Fisk JD. Anti-NR2 glutamate receptor antibodies and cognitive function in systemic lupus erythematosus. J Rheumatol. (2006) 33:1553-8.

32. Tsuchiya H, Haga S, Takahashi Y, Kano T, Ishizaka Y, Mimori A. Identification of novel autoantibodies to GABA(B) receptors in patients with neuropsychiatric systemic lupus erythematosus. Rheumatology (Oxford). (2014) 53:1219-28.

33. Magalhaes MB, da Silva LM, Voltarelli JC, Donadi EA, Louzada-Junior P. Lymphocytotoxic antibodies in systemic lupus erythematosus are associated with disease activity irrespective of the presence of neuropsychiatric manifestations. Scand J Rheumatol. (2007) 36:442-7.

34. Denburg SD, Behmann SA, Carbotte RM, Denburg JA. Lymphocyte antigens in neuropsychiatric systemic lupus erythematosus. Relationship of lymphocyte antibody specificities to clinical disease. Arthritis Rheum. (1994) 37:369-75.

35. Long AA, Denburg SD, Carbotte RM, Singal DP, Denburg JA. Serum lymphocytotoxic antibodies and neurocognitive function in systemic lupus erythematosus. Ann Rheum Dis. (1990) 49:249-53.

36. Sato S, Yashiro M, Asano T, Kobayashi H, Watanabe H, Migita K. Association of anti-triosephosphate isomerase antibodies with aseptic meningitis in patients with neuropsychiatric systemic lupus erythematosus. Clin Rheumatol. (2017) 36:1655-9.

37. Hanly JG, Hong C, White TD. Brain synaptosomal antibodies in systemic lupus erythematosus. Lupus. (1993) 2:35-45.

38. Ndhlovu M, Preuss BE, Dengjel J, Stevanovic S, Weiner SM, Klein R. Identification of alpha-tubulin as an autoantigen recognized by sera from patients with neuropsychiatric systemic lupus erythematosus. Brain Behav Immun. (2011) 25:279-85.

39. Iizuka N, Okamoto K, Matsushita R, Kimura M, Nagai K, Arito M, et al. Identification of autoantigens specific for systemic lupus erythematosus with central nervous system involvement. Lupus. (2010) 19:717-26.

40. Ikenouchi-Sugita A, Yoshimura R, Okamoto T, Umene-Nakano W, Ueda N, Hori H, et al. Serum brain-derived neurotrophic factor levels as a novel biological marker for the activities of psychiatric symptoms in systemic lupus erythematosus. World J Biol Psychiatry. (2010) 11:121-8.

41. Tamashiro LF, Oliveira RD, Oliveira R, Frota ER, Donadi EA, Del-Ben CM, et al. Participation of the neutrophin brain-derived neurotrophic factor in neuropsychiatric systemic lupus erythematosus. Rheumatology (Oxford). (2014) 53:2182-90.

42. Mader S, Jeganathan V, Arinuma Y, Fujieda Y, Dujmovic I, Drulovic J, et al. Understanding the Antibody Repertoire in Neuropsychiatric Systemic Lupus Erythematosus and Neuromyelitis Optica Spectrum Disorder: Do They Share Common Targets?. Arthritis Rheumatol. (2018) 70:277-286

43. Pröbstel AK, Thanei M, Erni B, Lecourt AC, Branco L, André R, Roux-Lombard P, et al. Association of antibodies against myelin and neuronal antigens with neuroinflammation in systemic lupus erythematosus. Rheumatology (Oxford). (2018) Sep 27.

44. Mikdashi J, Handwerger B. Predictors of neuropsychiatric damage in systemic lupus erythematosus: data from the Maryland lupus cohort. Rheumatology (Oxford). (2004) 43:1555-60.

45. Hu C, Huang W, Chen H, Song G, Li P, Shan Q, et al. Autoantibody profiling on human proteome microarray for biomarker discovery in cerebrospinal fluid and sera of neuropsychiatric lupus. PloS One. (2015) 10:e0126643.

46. Hanly JG, Urowitz MB, Su L, Bae SC, Gordon C, Clarke A, et al. Autoantibodies as biomarkers for the prediction of neuropsychiatric events in systemic lupus erythematosus. Ann Rheum Dis. (2011) 70:1726-32.

47. Karassa FB, Afeltra A, Ambrozic A, Chang DM, De Keyser F, Doria A, et al. Accuracy of anti-ribosomal P protein antibody testing for the diagnosis of neuropsychiatric systemic lupus erythematosus: an international meta-analysis. Arthritis Rheum. (2006) 54:312-24.

48. Hirohata S, Sakuma Y, Yanagida T, Yoshio T. Association of cerebrospinal fluid anti-Sm antibodies with acute confusional state in systemic lupus erythematosus. Arthritis Res Ther. (2014) 16:450.

49. Sato T, Fujii T, Yokoyama T, Fujita Y, Imura Y, Yukawa N, et al. Anti-U1 RNP antibodies in cerebrospinal fluid are associated with central neuropsychiatric manifestations in systemic lupus erythematosus and mixed connective tissue disease. Arthritis Rheum. (2010) 62:3730-40.

50. Margutti P, Sorice M, Conti F, Delunardo F, Racaniello M, Alessandri C, et al. Screening of an endothelial cDNA library identifies the C-terminal region of Nedd5 as a novel autoantigen in systemic lupus erythematosus with psychiatric manifestations. Arthritis Res Ther. (2005) 7:R896-903.

51. Cojocaru IM, Cojocaru M, Butnaru L, Miu G, Tanasescu R. Study of anti-endothelial cell antibodies in SLE patients with acute ischemic stroke. Rom J Intern Med. (2010) 48:173-7.

52. Conti F, Alessandri C, Bompane D, Bombardieri M, Spinelli FR, Rusconi AC, et al. Autoantibody profile in systemic lupus erythematosus with psychiatric manifestations: a role for anti-endothelial-cell antibodies. Arthritis Res Ther. (2004) 6:R366-72.

53. Sun XY, Shi J, Han L, Su Y, Li ZG. Anti-histones antibodies in systemic lupus erythematosus: prevalence and frequency in neuropsychiatric lupus. J Clin Lab Anal. (2008) 22:271-7.

54. van der Meulen PM, Barendregt AM, Cuadrado E, Magro-Checa C, Steup-Beekman GM, Schonenberg-Meinema D, et al. Protein array autoantibody profiles to determine diagnostic markers for neuropsychiatric systemic lupus erythematosus. Rheumatology (Oxford). (2017) 56:1407-16.

55. Schenatto CB, Xavier RM, Bredemeier M, Portela LV, Tort AB, Dedavid e Silva TL, et al. Raised serum S100B protein levels in neuropsychiatric lupus. Ann Rheum Dis. (2006) 65:829-31.

56. Yang XY, Lin J, Lu XY, Zhao XY. Expression of S100B protein levels in serum and cerebrospinal fluid with different forms of neuropsychiatric systemic lupus erythematosus. Clin Rheumatol. (2008) 27:353-7.

57. Fragoso-Loyo H, Cabiedes J, Atisha-Fregoso Y, Llorente L, Sanchez-Guerrero J. Utility of serum S100B protein for identification of central nervous system involvement in systemic lupus erythematosus. J Rheumatol. (2010) 37:2280-5.

58. Lapa AT, Postal M, Sinicato NA, Bellini BS, Fernandes PT, Marini R, et al. S100beta is associated with cognitive impairment in childhood-onset systemic lupus erythematosus patients. Lupus. (2017) 26:478-83.

59. Brunner HI, Klein-Gitelman MS, Zelko F, Beebe DW, Foell D, Lee J, et al. Blood-based candidate biomarkers of the presence of neuropsychiatric systemic lupus erythematosus in children. Lupus Sci Med. (2014) 1:e000038.

60. Ferreira I, Croca S, Raimondo MG, Matharu M, Miller S, Giles I, et al. Nitrated nucleosome levels and neuropsychiatric events in systemic lupus erythematosus; a multi-center retrospective case-control study. Arthritis Res Ther. (2017) 19:287.

61. Yajima N, Kasama T, Isozaki T, Odai T, Matsunawa M, Negishi M, et al. Elevated levels of soluble fractalkine in active systemic lupus erythematosus: potential involvement in neuropsychiatric manifestations. Arthritis Rheum. (2005) 52:1670-5.

62. Magro-Checa C, Schaarenburg RA, Beaart HJ, Huizinga TW, Steup-Beekman GM, Trouw LA. Complement levels and anti-C1q autoantibodies in patients with neuropsychiatric systemic lupus erythematosus. Lupus. (2016) 25:878-88.

63. Dellalibera-Joviliano R, Dos Reis ML, Cunha Fde Q, Donadi EA. Kinins and cytokines in plasma and cerebrospinal fluid of patients with neuropsychiatric lupus. J Rheumatol. (2003) 30:485-92.

64. Wang JB, Li H, Wang LL, Liang HD, Zhao L, Dong J. Role of IL-1beta, IL-6, IL-8 and IFN-gamma in pathogenesis of central nervous system neuropsychiatric systemic lupus erythematous. Int J Clin Exp Med. (2015) 8:16658-63.

65. Fragoso-Loyo H, Richaud-Patin Y, Orozco-Narvaez A, Davila-Maldonado L, Atisha-Fregoso Y, Llorente L, et al. Interleukin-6 and chemokines in the neuropsychiatric manifestations of systemic lupus erythematosus. Arthritis Rheum. (2007) 56:1242-50.

66. Lu XY, Zhu CQ, Qian J, Chen XX, Ye S, Gu YY. Intrathecal cytokine and chemokine profiling in neuropsychiatric lupus or lupus complicated with central nervous system infection. Lupus. (2010) 19:689-95.

67. Yoshio T, Okamoto H, Kurasawa K, Dei Y, Hirohata S, Minota S. IL-6, IL-8, IP-10, MCP-1 and G-CSF are significantly increased in cerebrospinal fluid but not in sera of patients with central neuropsychiatric lupus erythematosus. Lupus. (2016) 25:997-1003.

68. George-Chandy A, Trysberg E, Eriksson K. Raised intrathecal levels of APRIL and BAFF in patients with systemic lupus erythematosus: relationship to neuropsychiatric symptoms. Arthritis Res Ther. (2008) 10:R97.

69. Jara LJ, Irigoyen L, Ortiz MJ, Zazueta B, Bravo G, Espinoza LR. Prolactin and interleukin-6 in neuropsychiatric lupus erythematosus. Clin Rheumatol. (1998) 17:110-4.

70. Trysberg E, Carlsten H, Tarkowski A. Intrathecal cytokines in systemic lupus erythematosus with central nervous system involvement. Lupus. (2000) 9:498-503.

71. Jonsen A, Bengtsson AA, Nived O, Ryberg B, Truedsson L, Ronnblom L, et al. The heterogeneity of neuropsychiatric systemic lupus erythematosus is reflected in lack of association with cerebrospinal fluid cytokine profiles. Lupus. (2003) 12:846-50.

72. Asano T, Ito H, Kariya Y, Hoshi K, Yoshihara A, Ugawa Y, et al. Evaluation of blood-brain barrier function by quotient alpha2 macroglobulin and its relationship with interleukin-6 and complement component 3 levels in neuropsychiatric systemic lupus erythematosus. PloS One. (2017) 12:e0186414.

73. Hirohata S, Kanai Y, Mitsuo A, Tokano Y, Hashimoto H. Accuracy of cerebrospinal fluid IL-6 testing for diagnosis of lupus psychosis. A multicenter retrospective study. Clin Rheumatol. (2009) 28:1319-23.

74. Shiozawa S, Kuroki Y, Kim M, Hirohata S, Ogino T. Interferon-alpha in lupus psychosis. Arthritis Rheum. (1992) 35:417-22.

75. Fragoso-Loyo H, Atisha-Fregoso Y, Nunez-Alvarez CA, Llorente L, Sanchez-Guerrero J. Utility of interferon-alpha as a biomarker in central neuropsychiatric involvement in systemic lupus erythematosus. J Rheumatol. (2012) 39:504-9.

76. Hopia L, Thangarajh M, Khademi M, Laveskog A, Wallstrom E, Svenungsson E, et al. Cerebrospinal fluid levels of a proliferation-inducing ligand (APRIL) are increased in patients with neuropsychiatric systemic lupus erythematosus. Scand J Rheumatol. (2011) 40:363-72.

77. Iikuni N, Okamoto H, Yoshio T, Sato E, Kamitsuji S, Iwamoto T, et al. Raised monocyte chemotactic protein-1 (MCP-1)/CCL2 in cerebrospinal fluid of patients with neuropsychiatric lupus. Ann Rheum Dis. (2006) 65:253-6.

78. Ushigusa T, Ichinose K, Sato S, Michitsuji T, Shimizu T, Umeda M, et al. Soluble alpha-klotho is a potential biomarker associated with neuropsychiatric systemic lupus erythematosus. Clin Immunol. (2016) 165:29-34.

79. Trysberg E, Blennow K, Zachrisson O, Tarkowski A. Intrathecal levels of matrix metalloproteinases in systemic lupus erythematosus with central nervous system engagement. Arthritis Res Ther. (2004) 6:R551-6.

80. Ainiala H, Hietaharju A, Dastidar P, Loukkola J, Lehtimaki T, Peltola J, et al. Increased serum matrix metalloproteinase 9 levels in systemic lupus erythematosus patients with neuropsychiatric manifestations and brain magnetic resonance imaging abnormalities. Arthritis Rheum. (2004) 50:858-65.

81. Kwiecinski J, Klak M, Trysberg E, Blennow K, Tarkowski A, Jin T. Relationship between elevated cerebrospinal fluid levels of plasminogen activator inhibitor 1 and neuronal destruction in patients with neuropsychiatric systemic lupus erythematosus. Arthritis Rheum. (2009) 60:2094-101.

82. Karassa FB, Ioannidis JP, Touloumi G, Boki KA, Moutsopoulos HM. Risk factors for central nervous system involvement in systemic lupus erythematosus. QJM. (2000) 93:169-74.

83. Jongen PJ, Doesburg WH, Ibrahim-Stappers JL, Lemmens WA, Hommes OR, Lamers KJ. Cerebrospinal fluid C3 and C4 indexes in immunological disorders of the central nervous system. Acta Neurol Scand. (2000) 101:116-21.

84. Sanders ME, Alexander EL, Koski CL, Frank MM, Joiner KA. Detection of activated terminal complement (C5b-9) in cerebrospinal fluid from patients with central nervous system involvement of primary Sjogren's syndrome or systemic lupus erythematosus. J Immunol. (1987) 138:2095-9.
