# Supplementary material for: Laboratory and Neuroimaging Biomarkers in Neuropsychiatric Systemic Lupus Erythematosus: Where Do We Stand, Where To Go?
Source: Front Med (Lausanne). 2018 Dec 4;5:340. doi: 10.3389/fmed.2018.00340 (PMC6288259; doi:10.3389/fmed.2018.00340)
Supplement: Supplementary file 2 [file Data_Sheet_2.docx]

| **Supplementary Table 2** |
| --- |
| **Relaxation based MRI** |
| The two “classic” MRI relaxation times, T_1_, T_2_ are tightly linked to motility of water molecules in tissue and to exchange of water and water protons with e.g. macromolecular structures inside and outside cells. Whole brain maps of T_1_ and T_2_ values can be achieved using standard sequences available on all clinical MRI scanners. T_1_ has been solidly linked to the amount of myelin in a voxel (1), thus T_1_ maps are believed to quantitatively represent the degree of myelination across the brain (2, 3). T_2_ values significantly increase with the fraction of more mobile, or “free” water, for example in chronic ischemia and intracellular edema (4). |
| **Magnetization Transfer Imaging** |
| In a magnetization transfer imaging, the detectable MRI signal in an image is indirectly attenuated by saturating the undetectable signal originating from semi-solid tissue components, which in turn attenuates the tissue water signal via transfer of the saturated magnetization through chemical exchange or dipole-dipole interaction. Ratio with an image acquired without saturation provides the magnetization transfer ratio (MTR) image, a sensitive quantitative measure to alteration in tissue composition in disease (5).  This technique reflects in a quantitative way the integrity of macromolecular structures that exchange magnetization with the surrounding water and is sensitive to macroscopic and microscopic abnormalities. |
| **Diffusion Weighted Imaging and Diffusion Tensor Imaging** |
| This technique measures the microscopic motion of water protons. Differences in the magnitude of diffusion of water molecules in the brain, which depends on several known and unknown factors, can be translated into image contrast and quantitative brain maps. The rate of diffusion in living systems is referred to as the apparent diffusion coefficient (ADC) or mean diffusivity (MD). Molecular movement in tissues such as white matter is not the same in all directions, which is known as anisotropy. DTI analyses the three-dimensional shape of the diffusion. With this technique white matter fiber tracts can be reconstructed by combining the magnitude and directionality information of this anisotropic diffusion (6). The magnitude is represented by the MD while the directionality of water diffusion is represented by the fractional anisotropy (FA). Moreover, the rate of diffusion in the principal direction and the perpendicular direction to the white matter tract are called axial diffusivity and radial diffusivity (RD), respectively. All these parameters provide sensitive, but nonspecific measures of microstructural changes in brain tissue and are typically assessed in clinical studies by region-of-interest or voxel-based analysis (7). |
| **Perfusion based MRI** |
| Perfusion is the process of the delivery of blood to a capillary bed in tissue and therefore the passage of water and nutrients from the capillary bed to the brain parenchyma. Regional perfusion is intimately linked to regional blood flow and volume, and disease-related regional increases or decreases in perfusion are oftentimes indicative of active pathomechanism, such as inflammation or metabolic disturbance. Perfusion based MRI can be performed with the use of contrast agents, and can sensitively detect areas with compromised blood brain barrier or other disruptions in the microvasculature. Perfusion MRI without administration of a contrast agent is also possible, using the MRI to label the arterial blood at the neck level with a radiofrequency pulse. Such methods, called arterial spin labeling (ASL) techniques are less sensitive than those that use contrast agents, but benefit from non-invasiveness and from an improved selectivity of the perfused brain domains, based on the selected labeled artery (see reviews: (8, 9)). |
| **Magnetic Resonance Spectroscopy** |
| MR spectroscopy (MRS) detects the signal of protons of other species than water and map them across the brain. This results in concentration maps of a number of physiologically-relevant metabolites that can be modified by disease (10, 11). For example, disease-related modulation of the neuronal metabolite N-acetylasparate (NAA) is seen in neurodegenerative diseases (12, 13), and glial metabolites such as choline compounds (tCho) are modulated in neuroinflammation and more drammatically so in brain tumors (14, 15). |
| **Susceptibility based MRI** |
| MRI can be sensitized to the presence of substances with *high magnetic susceptibility* that cause small local disturbances to the local magnetic field and thus a decrease in signal in T_2_^*^-weighted MR image. A prime example is iron, which gives rise to strong contrast in iron-rich areas and also in large veins, where the high concentration of non-oxygenated hemoglobin is the source of contrast. This contrast has been harnessed to investigate the link between iron in the brain and pathology in a host of neurodegenerative and neurological disorders as well as to obtain crisp images of the brain vein network without the use of contrast agents. Recently, a quantitative link has been established between iron concentrations and the MR image *phase*. Quantitative susceptibility mapping (QSM) allows for better quantification of iron in the brain, also across MRI platforms and across magnetic fields (for a comprehensive review, see (16)). |

**REFERENCES**

1. Stüber C, Morawski M, Schäfer A, Labadie C, Wähnert M, Leuze C, et al. Myelin and iron concentration in the human brain: a quantitative study of MRI contrast. Neuroimage. (2014). 93:95-106.

2. Bock NA, Hashim E, Janik R, Konyer NB, Weiss M, Stanisz GJ, et al., Optimizing T1-weighted imaging of cortical myelin content at 3.0 T. Neuroimage. (2013). 65:1-12.

3. Dinse J, Härtwich N, Waehnert MD, Tardif CL, Schäfer A, Geyer S, et al. A cytoarchitecture-driven myelin model reveals area-specific signatures in human primary and secondary areas using ultra-high resolution in-vivo brain MRI. Neuroimage. (2015). 114:71-87.

4. Sibbitt WL Jr, Brooks WM, Haseler LJ, Griffey RH, Frank LM, Hart BL, et al., Spin-spin relaxation of brain tissues in systemic lupus erythematosus. A method for increasing the sensitivity of magnetic resonance imaging for neuropsychiatric lupus. Arthritis Rheum. (1995). 38: 810-8.

5. Grossman RI. Magnetization transfer in multiple sclerosis. Ann Neurol. (1994). 36: S97-9.

6. Basser PJ, Mattiello J, LeBihan D. Estimation of the effective self-diffusion tensor from the NMR spin echo. J Magn Reson B. (1994). 103:247-54.

7. Le Bihan D. Molecular diffusion, tissue microdynamics and microstructure. NMR Biomed. (1995). 8:375-86.

8. Detre JA, Alsop DC. Perfusion magnetic resonance imaging with continuous arterial spin labeling: methods and clinical applications in the central nervous system. Eur J Radiol. (1999). 30:115-24.

9. Barbier EL, Lamalle L, Decorps M. Methodology of brain perfusion imaging. J Magn Reson Imaging. (2001). 13:496-520.

10. Choi JK, Dedeoglu A, Jenkins BG. Application of MRS to mouse models of neurodegenerative illness. NMR Biomed. (2007). 20:216-37.

11. Hetherington HP, Pan JW, Chu WJ, Mason GF, Newcomer BR. Biological and clinical MRS at ultra-high field. NMR Biomed. (1997). 10:360-71.

12. McClure RJ, Kanfer JN, Panchalingam K, Klunk WE, Pettegrew JW. Magnetic resonance spectroscopy and its application to aging and Alzheimer's disease. Neuroimaging Clin N Am. (1995). 5:69-86.

13. Richards TL. Proton MR spectroscopy in multiple sclerosis: value in establishing diagnosis, monitoring progression, and evaluating therapy. AJR Am J Roentgenol. (1991). 157:1073-8.

14. Gupta RK, Sinha U, Cloughesy TF, Alger JR. Inverse correlation between choline magnetic resonance spectroscopy signal intensity and the apparent diffusion coefficient in human glioma. Magn Reson Med. (1999). 41:2-7.

15. Tzika AA, Astrakas LG, Zarifi MK, Petridou N, Young-Poussaint T, Goumnerova L, et al. Multiparametric MR assessment of pediatric brain tumors. Neuroradiology. (2003). 45:1-10.

16. Ghassaban K, Liu S, Jiang C, Haacke EM. Quantifying iron content in magnetic resonance imaging. Neuroimage. 2018.
