# Supplementary material for: Laboratory and Neuroimaging Biomarkers in Neuropsychiatric Systemic Lupus Erythematosus: Where Do We Stand, Where To Go?
Source: Front Med (Lausanne). 2018 Dec 4;5:340. doi: 10.3389/fmed.2018.00340 (PMC6288259; doi:10.3389/fmed.2018.00340)
Supplement: Supplementary file 3 [file Data_Sheet_3.docx]

**Panel 1.** Common lupus-prone strains used for modeling NP-SLE manifestations

***MRL/lpr* mice**

- Develop a severe form of the disease characterized by lymphoid hyperplasia, a wide range of autoantibodies (i.e. antibodies against DNA and brain antigens) and circulating immune complexes due to a mutation in the lpr (lympho-proliferative) gene on chromosome 19 and a dysfunctional Fas receptor.
- This defect results in a wide spectrum of autoimmune manifestations. NP manifestations appear usually early in these mice and include depression, anxiety, decreased locomotion and apathy. (1, 2, 3) In addition, neuropathological studies have found structural changes in the hippocampus, substantia nigra and brainstem.(2)

***NZB/NZW F1* mice**

- Develop a severe autoimmune disease characterized by elevated autoantibodies (i.e. antibodies against DNA and Sm), lymphadenopathy, splenomegaly, a severe immune-complex-mediated glomerulonephritis, due to genetic mutations among the genes in the major histocompatibility complex region.(4, 5)
- These mice present impairment in learning and mood-related behaviors developing late in the disease course. In addition, these NP manifestations correlate with a decreased level of several neuropeptides in the hippocampus, cortex and hypothalamus.(1, 6, 7)

***564Igi* lupus-prone mouse strain**

- A mice model that express an activation-induced cytidine deaminase transgene (*Aicda^tg^*), resulting in loss of tolerance expressing autoantibodies, mild peripheral inflammation and interferon-α receptor 1 (IFNAR)-dependent autoimmunity has become a successful tool for investigating NP-SLE.
- These mice develop a mild lupus-like disease and comparing with other models they develop pathology at 12 weeks after a normal adult period of 6-8 weeks.
- They present anxiety-like phenotypes, cognitive impairment and social interaction defects while no depression-like phenotypes or motor defects are found.(8)

**REFERENCES**

1. Jeltsch-David H, Muller S. Neuropsychiatric systemic lupus erythematosus and cognitive dysfunction: the MRL-lpr mouse strain as a model. Autoimmun Rev. (2014) 13:963-73.

2. Ballok DA, Earls AM, Krasnik C, Hoffman SA, Sakic B. Autoimmune-induced damage of the midbrain dopaminergic system in lupus-prone mice. J Neuroimmunol. (2004) 152:83-97.

3. Gulinello M, Putterman C. The MRL/lpr mouse strain as a model for neuropsychiatric systemic lupus erythematosus. J Biomed Biotechnol. (2011) 2011:207504.

4. Crampton SP, Morawski PA, Bolland S. Linking susceptibility genes and pathogenesis mechanisms using mouse models of systemic lupus erythematosus. Dis Model Mech. (2014) 7:1033-46.

5. Kotzin BL, Palmer E. Genetic contributions to lupus-like disease in NZB/NZW mice. The Am J Med. (1988) 85:29-31.

6. Kier AB. Clinical neurology and brain histopathology in NZB/NZW F1 lupus mice. J Comp Pathol. (1990) 102:165-77.

7. Bracci-Laudiero L, Aloe L, Lundeberg T, Theodorsson E, Stenfors C. Altered levels of neuropeptides characterize the brain of lupus prone mice. Neurosci Lett. (1999) 275:57-60.

8. Bialas AR, Presumey J, Das A, van der Poel CE, Lapchak PH, Mesin L, et al. Microglia-dependent synapse loss in type I interferon-mediated lupus. Nature. (2017) 546:539-43.
